# Vegetation-Specific Cooling Responses to Compact Urban Development: Evidence from a Landscape-Based Analysis in Nanjing, China

**DOI:** 10.3390/plants14162457

**Published:** 2025-08-08

**Authors:** Qianyu Sun, Daicong Li, Xiaolan Tang, Yujie Ren

**Affiliations:** 1 Department of Urban and Rural Planning, Nanjing Forestry University, Nanjing 210037, China; sqy09_19@163.com (Q.S.);; 2 Institute of Ecological Civilization Construction and Forestry Development, Nanjing Forestry University, Nanjing 210037, China

**Keywords:** urban vegetation, vegetation cooling effect, compact urban development, urban greening strategies

## Abstract

The urban heat island (UHI) effect has emerged as a growing ecological challenge in compact urban environments. Although urban vegetation plays a vital role in mitigating thermal extremes, its cooling performance varies depending on vegetation type and urban morphological context. This study explores the extent to which compact urban development—quantified using the Mixed-use and Intensive Development (MIXD) index—modulates the cooling responses of different vegetation types in Nanjing, China. A combination of landscape metrics, regression-based interaction models, and XGBoost with SHAP analysis is employed to uncover vegetation-specific and structure-sensitive cooling effects. The results indicate that densely planted trees exhibit reduced cooling effectiveness in compact areas, where spatial clustering and fragmentation tend to intensify UHI effects, particularly during nighttime. In contrast, scattered trees are found to maintain more stable cooling performance across varying degrees of urban compactness, while low-lying vegetation demonstrates limited thermal regulation capacity. Critical thresholds of MIXD (approximately 28 for UHI area and 37 for UHI intensity) are identified, indicating a nonlinear modulation of green space performance. These findings underscore the importance of vegetation structure and spatial configuration in shaping urban microclimates and offer mechanistic insights into plant–environment interactions under conditions of increasing urban density.

## 1. Introduction

As cities pursue increasingly ambitious sustainability goals, mitigating the Urban Heat Island (UHI) effect has become a critical imperative for promoting ecological resilience, public health, and environmental justice [1,2]. UHI describes the phenomenon whereby urban areas exhibit elevated surface and air temperatures compared to surrounding rural regions, largely due to intensified built density, extensive impervious surfaces, reduced vegetation cover, and anthropogenic heat emissions [3,4]. Urban green infrastructure—including parks, street trees, and green roofs—provides crucial thermal relief through shading, evapotranspiration, and enhanced surface albedo [5]. Beyond cooling, such nature-based solutions support biodiversity conservation, carbon sequestration, and improved environmental quality, directly contributing to the attainment of multiple United Nations Sustainable Development Goals (SDGs), particularly SDG 11 on sustainable cities and communities [6,7].

Although a wealth of empirical studies has confirmed the capacity of urban vegetation to mitigate UHI [8,9], its effectiveness is not uniform across urban environments. The accelerating shift toward Compact Urban Development—defined by high-density built forms, vertical intensification, and mixed land-use—has introduced increasingly complex spatial contexts that may fundamentally reshape the way vegetation interacts with urban microclimates [10]. While compact urban form is widely promoted for its benefits in curbing urban sprawl and improving land-use efficiency, its implications for climate adaptation remain understudied. Specifically, current research has seldom investigated how compact morphology modulates the cooling performance of green spaces, or whether these effects are mediated by specific spatial patterns and landscape structures [11,12]. This gap hinders the development of robust greening strategies that remain effective under conditions of urban densification.

To address this critical knowledge gap, the present study investigates the influence of Compact Urban Development on the cooling efficacy of urban vegetation, using Nanjing, China, as a representative high-density city undergoing rapid transformation. The following research questions are posed: under what conditions are vegetation-based thermal mitigation amplified or constrained by compact urban morphology? Are spatial heterogeneities or nonlinear thresholds present in this relationship that merit consideration in urban climate policy and spatial design?

A hybrid methodological approach is employed, combining spatial regression models with interpretable machine learning techniques—specifically, XGBoost and SHAP—to identify both generalizable trends and localized variations. In addition, landscape metrics, including Percentage of Landscape (PLAND), Euclidean Nearest Neighbor Distance (ENN), and Landscape Shape Index (LSI), are incorporated to capture key structural attributes of urban greenery. This integrated framework extends beyond conventional greenness indices by explicitly modeling the interactions between built morphology and ecological configurations.

The findings of this study contribute to both theoretical and practical domains. From a theoretical perspective, the results demonstrate that vegetation’s cooling capacity is conditionally shaped by urban morphological characteristics, thereby advancing the understanding of form-dependent contingencies in urban climate resilience. From a practical standpoint, the outcomes provide actionable insights for designing climate-adaptive green infrastructure in high-density, functionally heterogeneous urban environments. These contributions are particularly relevant for rapidly urbanizing regions in the Global South, where balancing land-use intensification with thermal livability remains a pressing planning challenge.

## 2. Results

This section presents the empirical results addressing the following central research question: how does compact urban development influence the capacity of vegetation to mitigate urban heat? Urban compactness is quantified using a composite index of mixed-use intensity and built density (MIXD), and its interactions with key structural attributes of urban greenery—specifically, PLAND, ENN, and LSI—are examined across different vegetation types. By incorporating interaction terms between MIXD and vegetation metrics, the moderating effects of compact urban form on the thermal performance of densely planted trees, scattered trees, and low-lying vegetation are isolated. In addition, the regression-based findings are complemented by machine learning models to identify threshold values of MIXD at which shifts in cooling effectiveness become pronounced. This multi-method approach facilitates a nuanced assessment of how vegetation structure and urban morphology jointly shape thermal outcomes in compact city environments.

### 2.1. Evaluating Thermal Mitigation Performance of Urban Vegetation Types

#### 2.1.1. Vegetation Coverage and Morphology as Determinants of UHI Presence

The influence of vegetation structure on the likelihood of UHI occurrence during both daytime and nighttime was first examined. Logistic regression models were estimated using the binary presence of UHI as the dependent variable, with PLAND, ENN, and LSI as independent variables representing three vegetation types: densely planted trees, scattered trees, and low-lying vegetation. Impervious surface ratio and building density were included as control variables. The results are presented in Table 1.

Both models achieved satisfactory explanatory performance, with pseudo R^2^ values of 47.26% (daytime) and 44.03% (nighttime), and statistically significant likelihood ratio tests (*p* < 0.001). PLAND consistently displayed significant negative associations with UHI presence across all vegetation types. The strongest association was observed for scattered trees during daytime (β = −13.00, *p* < 0.05), followed by low-lying vegetation and densely planted trees. ENN showed significant negative coefficients for densely planted trees and low-lying vegetation, indicating that greater patch proximity is associated with lower UHI probability for these types. In contrast, ENN for scattered trees was not significant in either model. LSI exhibited a positive and significant association with UHI presence for densely planted trees and low-lying vegetation, while effects for scattered trees were non-significant.

Taken together, these findings identify vegetation coverage as the most consistent predictor of UHI presence reduction. Effects related to spatial proximity and shape complexity vary by vegetation type, particularly between aggregated and dispersed forms.

#### 2.1.2. Vegetation Structure as a Driver of UHI Spatial Extent and Intensity

To evaluate the influence of vegetation structure on the spatial magnitude and severity of urban heat, linear regression models were employed with daytime and nighttime UHI area and intensity as dependent variables. The same set of landscape metrics—PLAND, ENN, and LSI—was used as predictors for each vegetation type. All models were found to be statistically significant, with those predicting UHI area demonstrating higher explanatory power (R^2^ = 68.85% and 66.16% for daytime and nighttime, respectively) compared to the models for UHI intensity (R^2^ = 47.06% and 35.99%) (Table 2).

Vegetation coverage emerged as the most consistent and robust predictor across all models. For example, increasing PLAND in densely planted trees was significantly associated with reductions in both UHI area and intensity during the daytime (e.g., β = –5.03 and –0.72, respectively; *p* < 0.05), with similar though weaker effects observed for scattered trees and low-lying vegetation. This confirms that vegetation extent plays a foundational role in surface cooling, regardless of vegetation form.

The spatial configuration of vegetation, however, revealed more nuanced effects. For densely planted trees, lower ENN—indicating tighter spatial clustering—was linked to significantly reduced daytime UHI magnitude (β = –124.74 for area, β = –17.08 for intensity; *p* < 0.05). A similar, though less pronounced, pattern was observed for low-lying vegetation. In contrast, scattered trees showed a positive association between ENN and nighttime UHI, suggesting that excessive clustering may reduce their cooling potential under certain conditions.

The influence of LSI varied notably across vegetation types. In densely planted trees, higher LSI (i.e., more irregular shapes) was significantly associated with greater UHI area and intensity during the day, whereas in scattered trees, the opposite pattern was observed—lower LSI values were linked to improved cooling outcomes. For low-lying vegetation, the effect of LSI was weak and statistically insignificant, indicating limited sensitivity to shape complexity.

Taken together, these results demonstrate that vegetation-mediated cooling is not solely a function of coverage, but also of how green elements are spatially organized. The effectiveness of structural attributes such as clustering and shape regularity varies by vegetation type and time of day, underscoring the need to tailor urban greening strategies to context-specific landscape configurations.

### 2.2. Moderating Role of Compact Urban Development

#### 2.2.1. Compact Urban Development as a Moderator of UHI Suppression

To evaluate whether compact urban form modulates the thermal performance of vegetation, interaction terms between the MIXD index and vegetation structure metrics (PLAND, ENN, LSI) were incorporated into logistic regression models predicting the presence of UHI during both daytime and nighttime. This modeling strategy enables a vegetation-type-specific assessment of how spatial compactness alters the effectiveness of green infrastructure in mitigating urban heat. The corresponding results are reported in Table 3.

The models retained high explanatory power, with pseudo R^2^ values of 47.19% for daytime and 43.47% for nighttime, and highly significant likelihood ratio tests (*p* < 0.001). These values are comparable to the baseline models, indicating that the inclusion of compactness interactions does not compromise model stability and allows meaningful interpretation of moderating effects.

Across vegetation types, responses to compact urban form were uneven. For densely planted trees, the interaction between MIXD and ENN was positively associated with nighttime UHI presence (β = 363.26, *p* < 0.1), contrasting with the baseline cooling pattern of clustered canopies. This suggests that in compact settings, dense vegetative aggregation may coincide with residual heat retention rather than alleviation. A similar reversal was observed for PLAND, where its interaction with MIXD was positively related to daytime UHI presence (β = 0.0036, *p* < 0.1), implying that increased vegetation coverage alone may not guarantee thermal mitigation under spatial constraints.

Fragmentation effects also appeared contingent on urban form. Significant negative interactions between MIXD and LSI were observed for densely planted trees and low-lying vegetation (*p* < 0.1), indicating that irregular shapes may further diminish the thermal benefits of vegetation in compact zones. In contrast, scattered trees consistently showed no significant interactions with MIXD across all three structural dimensions, reinforcing their relative insensitivity to compactness-related structural shifts.

Taken together, these results suggest that compact urban development does not uniformly reduce vegetation effectiveness but selectively alters the influence of structure and form. Densely planted trees emerge as the most sensitive to compactness—particularly in terms of spatial cohesion and fragmentation—while scattered trees demonstrate structural stability. Recognizing these differentiated effects is critical to adapting vegetation strategies for heat mitigation in densifying urban environments.

#### 2.2.2. Compact Urban Development and Vegetation Responses in UHI Extent and Intensity

To investigate whether compact urban form alters the magnitude of vegetation-based thermal mitigation, the baseline linear regression models were extended by incorporating interaction terms between the MIXD index and vegetation structure metrics. These models predicted UHI area and intensity for both daytime and nighttime periods, enabling a detailed assessment of how vegetation responses vary across thermal dimensions under differing urban form conditions. The corresponding results are presented in Table 4. Model performance remained satisfactory, with R^2^ values of 70.66% and 45.49% for UHI area, and 67.87% and 36.01% for UHI intensity, indicating substantial explanatory power—particularly during the daytime.

Results suggest that compactness affects vegetation types in distinct and sometimes opposing ways. Densely planted trees exhibited vulnerability to compact urban form; increased clustering under high MIXD conditions was associated with higher daytime UHI intensity (β = 42.15, *p* < 0.1), and fragmentation further amplified UHI area during both day and night (β ≈ 0.0056, *p* < 0.05). These findings point to a structural sensitivity, where aggregation and irregularity may reduce cooling performance in constrained urban contexts.

Scattered trees showed greater structural resilience. Neither clustering nor fragmentation significantly altered their cooling effect on UHI intensity. However, more regular shapes (i.e., lower LSI) were linked to reduced UHI area in compact zones (β = −0.0334, *p* < 0.05), suggesting that simplified spatial forms enhance thermal performance even in dense settings.

Low-lying vegetation revealed mixed patterns. While spatial proximity under compact form reduced daytime UHI intensity (β = −22.35, *p* < 0.05), its coverage was positively associated with UHI intensity (β = 0.0025, *p* < 0.05), indicating potential inefficiencies in ground-level vegetation expansion when ventilation and shading are limited.

Coverage effects also varied by vegetation type. For densely planted and scattered trees, increased canopy coverage remained beneficial in compact environments (e.g., MIXD × PLAND for DenseTree: β = −0.0028, *p* < 0.05), underscoring the value of extending tree cover when spatial integrity is preserved.

Overall, these findings confirm that compact urban development not only alters the structural composition of green space but also conditions its thermal outcomes. Vegetation type, spatial arrangement, and development intensity jointly shape the extent to which cooling benefits can be realized, emphasizing the need for tailored greening strategies in dense urban environments.

Taken together, the regression results show that vegetation-related cooling in Nanjing is governed by two interacting dimensions: how much vegetation is present and how that vegetation is spatially organized within varying degrees of compact urban development. In the baseline models (Section 2.1), greater coverage consistently corresponded to lower UHI occurrence, areal extent, and intensity across all vegetation types, with effects strongest for tree cover. Spatial metrics added nuance; closer patch proximity (low ENN) and lower shape irregularity (low LSI) supported reduced UHI for densely aggregated vegetation, whereas scattered tree cover was comparatively insensitive to these structural attributes. When MIXD was introduced (Section 2.2), these relationships proved conditional. Dense tree cover—highly effective under baseline conditions—became less reliable in compact settings; higher MIXD weakened the benefits of both coverage and clustering and amplified the penalty of fragmentation, particularly for nighttime UHI occurrence and daytime intensity. Scattered trees remained largely stable across MIXD gradients, with only modest evidence that simpler shapes improved performance in denser areas. Low-lying vegetation showed mixed behavior; compactness enhanced the benefit of spatial proximity for reducing daytime intensity, yet higher coverage under high MIXD aligned with higher heat levels, indicating diminishing returns at ground layer. Overall, compact morphology selectively constrains vegetation-based thermal mitigation where cooling depends on structural cohesion (dense canopies) and may even invert expected benefits when coverage expands without spatial integrity; dispersed tree structures are comparatively robust. These conditional patterns motivate the nonlinear threshold analysis presented in Section 2.3.

### 2.3. Identifying Nonlinear Thresholds of MIXD Influencing Vegetation Cooling

The prior regression results (Section 2.2) demonstrated that compact urban development significantly moderates the thermal performance of vegetation across three UHI dimensions. However, these moderation patterns may exhibit nonlinearity. To evaluate this possibility, a nonlinear machine learning approach (XGBoost) was employed to examine whether threshold effects exist in the relationship between MIXD and UHI mitigation. The analysis was conducted in two steps: Section 2.3.1 assesses model performance and feature importance, while Section 2.3.2 identifies critical MIXD thresholds beyond which cooling benefits either sharply decline or stabilize.

#### 2.3.1. Model Performance and Feature Importance

To elucidate how compact urban development influences the thermal regulation capacity of different vegetation types, three XGBoost models were trained to predict the following: (1) the extent of daytime UHI, (2) the intensity of daytime UHI, and (3) the extent of nighttime UHI. This modeling strategy builds directly upon the linear regression findings reported in Section 2.2, which identified significant moderation effects of MIXD on these three UHI dimensions. Accordingly, the XGBoost models were designed to further explore the nonlinear patterns and threshold behaviors associated with vegetation–compactness interactions. Each model incorporated green space structural indicators (PLAND, ENN, LSI) stratified by vegetation type, the MIXD index, and a suite of built-environment and socio-spatial control variables.

The models yielded generally robust predictive outcomes (Table 5), particularly in explaining spatial thermal patterns. The daytime UHI area model achieved an R^2^ of 0.76 and an RMSE of 1.43 km^2^, demonstrating strong explanatory capacity. The nighttime UHI area model followed with an R^2^ of 0.58, while the daytime UHI intensity model showed moderate performance (R^2^ = 0.37), likely reflecting the greater influence of transient microclimatic factors such as solar exposure and shading dynamics on thermal intensity compared to spatial extent.

**Figure 1:**
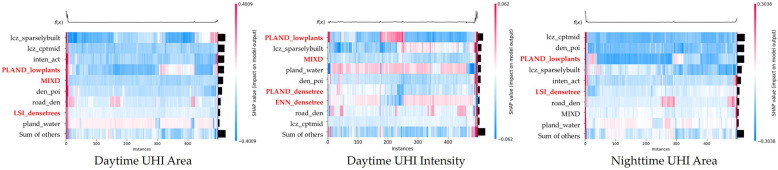
Ranking of feature importance.

Importantly, the ranking of feature importance across models highlights a consistent set of influential predictors (Figure 1), revealing critical vegetation–urban form interactions. Low-lying vegetation coverage (PLAND_LowPlants) emerged as a dominant factor across all models, confirming its stable and widespread cooling contribution—especially in dense urban fabrics. The MIXD index, while initially a moderating variable, also surfaced as a top-ranking direct predictor in both the daytime intensity and nighttime area models, reinforcing the hypothesis that urban compactness fundamentally reshapes the microclimatic efficacy of green infrastructure. Built-environment indicators, such as compact mid-rise building share (lcz_cptmid), sparsely built areas (lcz_sparselybuilt), and POI density, consistently ranked among the top contributors, suggesting that vegetation’s cooling performance is tightly coupled with the surrounding morphological and functional intensity. Notably, landscape structural indicators such as LSI and ENN of dense trees also entered the top-ten ranks, albeit with relatively lower importance values, implying that vegetation form remains relevant—but context-dependent—within thermally stressed urban settings.

Collectively, these findings establish a data-driven basis for exploring the nonlinear modulation of green space cooling potential under varying levels of urban compactness. The predictive strength of MIXD and its interactions with vegetation traits affirm the necessity of moving beyond linear models and toward threshold-based, adaptive planning frameworks—discussed in detail in Section 2.3.2.

#### 2.3.2. Optimal Urban Compactness for Vegetation-Based UHI Reduction

**Figure 2:**
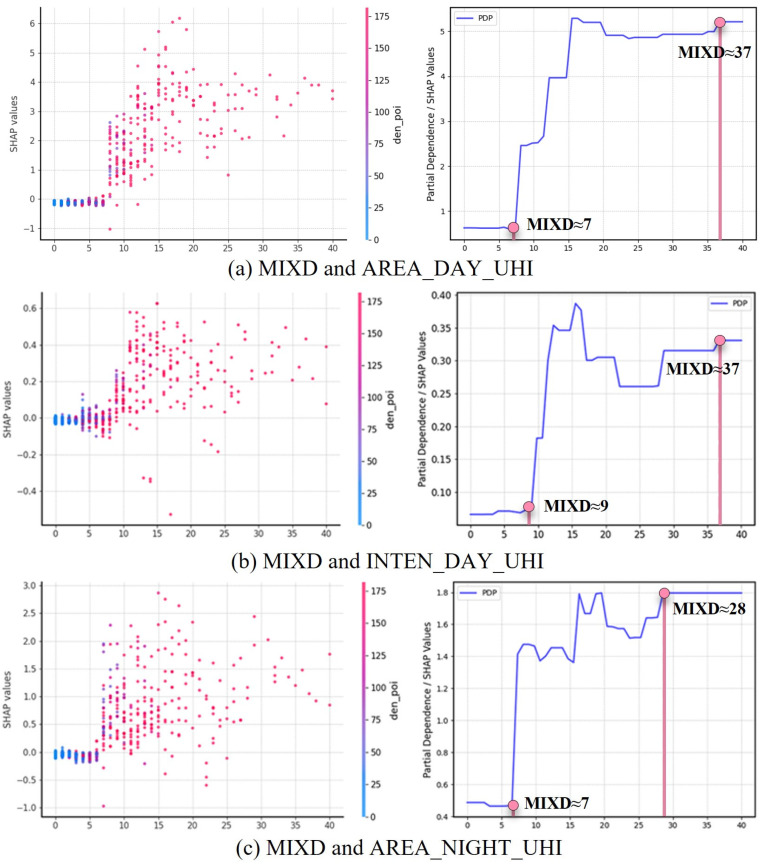
Partial dependence plots showing the nonlinear influence of MIXD on UHI metrics.

Building on the identification of the most relevant predictors for UHI mitigation in Section 2.3.1, this section further investigates the nonlinear relationships between MIXD and UHI mitigation outcomes, with a specific focus on how varying levels of land-use compactness influence the cooling effects of vegetation. Drawing from the prior moderation analyses, which indicated strong interactive effects of MIXD on three thermal metrics—daytime UHI area, nighttime UHI area, and daytime UHI intensity—three XGBoost models were constructed to assess the nonlinear influence of MIXD under different vegetation configurations. Partial dependence plots (PDPs) derived from SHAP analysis reveal distinct threshold behaviors across all three target outcomes (Figure 2).

For daytime UHI area, the PDP reveals a clear nonlinear escalation in thermal burden as MIXD increases. When MIXD values remain below 7, UHI area stays low and stable (~0.5 km^2^), indicating minimal compactness-related thermal amplification. However, between MIXD values of 7 and 15, UHI area expands rapidly from ~0.5 to ~5.1 km^2^, suggesting that this range constitutes a critical threshold where compact development begins to undermine vegetation cooling. Beyond MIXD ≈ 37, the curve flattens, and UHI area stabilizes despite further compactness increases, indicating saturation.

A similar pattern emerges for daytime UHI intensity, though with a slightly shifted threshold. Below MIXD ≈ 9, UHI intensity remains near baseline (~0.04 °C). Between MIXD 9 and 12.5, intensity increases sharply to ~0.35 °C, followed by moderate fluctuations up to MIXD ≈ 22. Another inflection point occurs between MIXD 22 and 37, culminating in a plateau beyond MIXD ≈ 37, where additional compactness no longer intensifies daytime heat, but cooling potential remains diminished.

For nighttime UHI area, the response curve again demonstrates nonlinear growth. The metric remains steady (~0.45 km^2^) when MIXD is below 6. However, from MIXD 6 to 16, UHI area increases substantially, peaking near 1.8 km^2^—likely reflecting delayed heat release and obstructed nocturnal ventilation. Beyond MIXD ≈ 28, the curve stabilizes, suggesting diminished sensitivity to further compactness.

Across all three models, the presence of turning points and saturation zones confirms the existence of threshold effects in the relationship between urban compactness and green infrastructure performance. These nonlinear analyses jointly suggest that the capacity of vegetation to mitigate urban heat is highly sensitive to the degree of land-use compactness. Across all three UHI metrics, a MIXD value below approximately 7 emerges as the most favorable range, within which vegetation retains strong thermal regulatory functions and urban densification poses minimal disruption. In contrast, the range of MIXD ≈ 7–15 represents a critical threshold zone, where sharp increases in both UHI area and intensity suggest a rapid degradation of green space effectiveness. Beyond MIXD ≈ 28–37, the thermal burden plateaus, but vegetation-based cooling remains substantially suppressed, indicating persistent structural constraints. These findings suggest that vegetation-based cooling does not decline linearly with urban densification; instead, certain ranges of MIXD are associated with disproportionately large losses in cooling efficiency. This supports the view that climate-sensitive urban design must consider nonlinear morphological constraints in addition to total green coverage.

## 3. Discussion

### 3.1. Cooling Mechanisms and Comparative Insights from Prior Research

This study underscores the complex pathways through which compact urban development modulates the cooling performance of different vegetation types. Contrary to the prevailing assumption that dense tree cover uniformly provides the most effective thermal mitigation, the results indicate that this benefit is significantly diminished in highly compact urban areas. Specifically, the regression models presented in Section 2.2 show that densely planted trees (DenseTree) exhibit reduced cooling efficiency—particularly in terms of UHI area and intensity—when situated within compact morphologies characterized by high MIXD values. These findings are consistent with previous research suggesting that compact urban form may obstruct airflow, increase anthropogenic heat accumulation, and induce canopy overshading, thereby limiting the potential for evapotranspiration and radiative heat loss [13,14].

Scattered trees, however, show more consistent cooling performance across compactness gradients. Their open and modular spatial arrangement may reduce local thermal inertia while facilitating air circulation, echoing the observations that emphasized the functional resilience of dispersed vegetation types in high-density zones [15,16]. Low-lying vegetation types also displayed conditional responses; while their area-based metrics (PLAND) negatively interacted with MIXD in some models, under certain configurations they exhibited enhanced performance, potentially due to unobstructed solar access and reduced edge effects—an insight that nuances previous understandings of low vegetation as merely supplementary to trees [17].

Notably, the structural indices of vegetation—PLAND, ENN, and LSI—emerged as key moderators in explaining the observed differences. For instance, higher edge density and fragmentation were consistently associated with weakened vegetation-induced cooling, particularly in the cases of DenseTree and LowPlant, suggesting that spatial integrity plays a critical role in landscape-scale temperature regulation. These findings reinforce principles from landscape ecology, which emphasize the importance of cohesive vegetative patches with minimal fragmentation for optimizing urban thermal mitigation [18]. Importantly, these results diverge from previous studies that treat urban compactness as a static background variable. In contrast, MIXD is shown to function as a dynamic moderator that influences the thermal effects of vegetation through both morphological (e.g., density, patch shape) and functional (e.g., land-use type, imperviousness) dimensions. This perspective complements and extends recent research advocating for more integrative metrics of urban form in climate-sensitive planning efforts [19,20].

### 3.2. Planning Implications for Threshold-Sensitive Urban Cooling

The identification of nonlinear thermal responses to compact urban development offers a valuable foundation for rethinking how green infrastructure should be designed and implemented under different urban morphologies. Instead of treating compactness as a uniform constraint, planning strategies should respond to its varying effects across the compactness continuum (Table 6).

For low-compactness zones (MIXD < 7), vegetation cooling performance remains relatively stable, suggesting that moderate densification is feasible without substantial thermal penalties. In such areas, planners may focus on increasing functional density while safeguarding existing green space, ensuring that new development does not fragment vegetative patches or create abrupt transitions in surface temperature [21]. Compact but permeable configurations—such as narrow streets lined with scattered trees and low-rise mixed-use buildings—can promote climate efficiency without suppressing cooling potential [22].

In moderate-compactness zones (MIXD ≈ 9–28), however, the results signal a critical threshold where green space efficiency declines rapidly. This transitional regime demands proactive thermal governance. Planning interventions should prioritize the continuity and connectivity of vegetation—especially for densely planted trees, which are vulnerable to performance loss under fragmented conditions [23]. The implementation of ventilation corridors, street canyon optimization, and rooftop greening systems can help restore evapotranspiration and enhance air exchange [24]. Moreover, landscape-level zoning that clusters low-lying vegetation and scattered trees in heat-prone neighborhoods may offer stable cooling benefits, particularly when combined with permeable pavement and low-reflectance materials [25].

In high-compactness zones (MIXD > 37), where UHI metrics plateau, vegetation performance is constrained not by density per se, but by microclimatic pressures such as low wind speed, limited soil exposure, and excessive shading [26]. Here, the emphasis should shift to vertical greening solutions, including façade vegetation, trellises, and modular planting systems [27]. These approaches are particularly suited to high-rise environments, where ground-level space is limited but thermal stress is intense. Integrating three-dimensional greening into building codes and incentive schemes can help institutionalize these solutions [28]. Importantly, the planning logic should be MIXD-sensitive, aligning greening strategies with the spatial realities of compact development rather than pursuing a uniform green coverage target [29]. Urban greenery cannot function optimally if deployed indiscriminately across diverse morphologies [30]. Instead, effective planning requires threshold-informed design [31], where spatial compactness guides both the type and arrangement of vegetation [32].

Finally, the presence of nonlinear response curves indicates that early interventions in moderately compact areas may yield disproportionately large benefits, while late-stage adjustments in highly dense cores may only marginally improve thermal conditions. This underscores the importance of spatial timing in planning, suggesting that cities should prioritize adaptive strategies in emerging or transforming neighborhoods, where morphology is still flexible.

### 3.3. Study Limitations and Future Research Directions

While this study provides an integrative view of how compact urban development modulates the cooling functions of green infrastructure, several limitations warrant attention and open pathways for future research.

One key limitation of this study lies in the spatial resolution adopted for the analysis. The use of 3 km × 3 km grid cells facilitated the detection of city-wide spatial structures and helped reduce noise in the calculation of landscape metrics. However, this coarse granularity may obscure fine-scale microclimatic processes that operate at the street or neighborhood level. Elements such as building orientation, façade shading, canyon ventilation, and vegetation–built interfaces are likely to exert critical but highly localized effects on urban heat regulation. Future research would benefit from the use of high-resolution spatial units—potentially derived from building footprint data, LiDAR, or 3D urban morphology—in order to more accurately capture the heterogeneity of urban thermal environments.

A second limitation concerns the temporal scope of the analysis, which is based on a single-year snapshot. This restricts the capacity to examine seasonal dynamics or inter-annual variability in the cooling performance of vegetation. Given that land surface temperature is influenced by factors such as vegetation phenology, solar angle, and anthropogenic heat emissions—all of which vary over time—longitudinal datasets and time-series remote sensing approaches could help reveal how compactness–vegetation interactions evolve under changing thermal conditions.

A further limitation relates to the conceptual and empirical validation of the MIXD index, used here as a core measure of compact urban form. While MIXD effectively integrates land-use mix and development intensity, it does not directly incorporate explicit morphological parameters such as floor area ratio (FAR), land occupation index (LOI), or building height—all of which are known to shape thermal and ventilation regimes. Moreover, the index has yet to be benchmarked against established urban form indicators. Future work should explore how MIXD compares with these conventional metrics in predicting UHI outcomes, and whether a hybrid or multi-indicator framework would enhance explanatory power and planning relevance.

In sum, addressing these limitations calls for the development of multi-scale, temporally dynamic, and morphologically rich analytical frameworks. As cities continue to densify, understanding how specific compactness configurations interact with vegetation structure across space and time will be essential for designing thermally resilient and ecologically effective urban environments.

## 4. Materials and Methods

### 4.1. Study Area

This study was conducted in Nanjing and its surrounding metropolitan region, located in Jiangsu Province, eastern China. As a core city within the Yangtze River Delta urban agglomeration, Nanjing has undergone rapid urbanization in recent decades, resulting in a typical pattern of compact, high-density development [33]. The region experiences a humid subtropical climate with hot summers and exhibits a pronounced UHI effect, making it a representative case for investigating the thermal modulation capacity of urban vegetation under intensifying land-use compactness [34]. Despite ongoing densification, Nanjing retains a relatively rich green infrastructure network, including large public parks, street-level greenery, and residential plantings. This co-occurrence of urban density and ecological assets provides a unique context for examining how different vegetation types interact with compact urban form to influence UHI dynamics.

**Figure 3:**
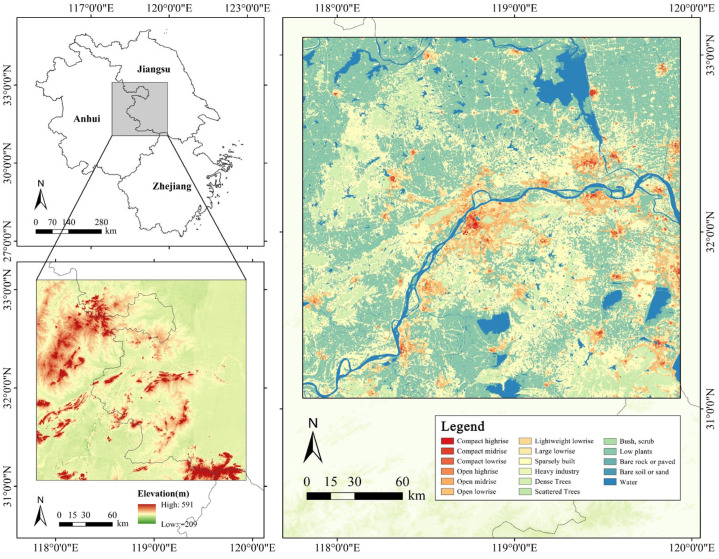
Study area.

For spatial analysis, the study area was partitioned into 4828 equal-sized grid cells with a spatial resolution of 0.03° × 0.03° (approximately 3.3 km × 3.3 km). This resolution was selected as a balanced scale that captures meso-level urban thermal and vegetation patterns while minimizing local heterogeneity and computational noise [35]. It is sufficiently fine to detect spatial variations in green space configuration and UHI characteristics across the urban landscape, yet coarse enough to ensure data comparability across multiple sources with varying spatial resolutions. Each grid served as the unit of analysis for extracting landscape metrics related to vegetation structure, UHI intensity and extent, and mixed-use development, thereby ensuring consistency across datasets and enhancing analytical robustness. An overview of the study area is shown in Figure 3.

### 4.2. Data Sources and Processing

#### 4.2.1. Landsat 8 TIRS: Urban Heat Island Metrics

Urban thermal characteristics were derived from the YCEO Global Pixel-Level Surface Urban Heat Island (SUHI) dataset, which provides annual summertime daytime and nighttime SUHI metrics at a 300 m resolution for over 10,000 urban agglomerations globally. This dataset is constructed using MODIS 8-day Land Surface Temperature (LST) products from Terra (~10:30 AM) and Aqua (~1:30 AM) satellites, in combination with LandScan urban extent, ESA CCI land cover, and GMTED2010 elevation data. The SUHI intensity was calculated using a standardized urban–rural LST difference algorithm, offering globally consistent measures of urban heat intensity without the need for scene-specific LST retrieval or post-classification [36].

To support spatially explicit analysis, three thermal indicators were extracted for each 0.03° × 0.03° grid cell during both daytime and nighttime periods: (1) UHI occurrence: a binary variable indicating the presence or absence of UHI, (2) UHI area: the proportion of UHI area in each grid, and (3) UHI intensity: the mean LST anomaly relative to the regional background. Although traditional methods employing Landsat 8 Thermal Infrared Sensor (TIRS) imagery and mono-window algorithms offer higher spatial resolution, the YCEO SUHI dataset provides harmonized global coverage and incorporates atmospheric correction, emissivity estimation, and temporal compositing at the data source. This makes it particularly well-suited for large-scale comparative analyses across heterogeneous urban environments, ensuring methodological consistency and minimizing processing bias. Widely adopted in recent urban climate and environmental studies, this globally harmonized dataset offers consistent SUHI metrics suitable for inter-city comparative research [37,38,39].

#### 4.2.2. LCZ Dataset: Urban Green Space Typology and Spatial Metrics

Urban green space typologies were identified based on the Global Local Climate Zone (LCZ) dataset developed by Demuzere et al. [40], available on Google Earth Engine (GEE), with a native spatial resolution of 100 m. This dataset, derived from remote sensing and crowdsourced features, classifies urban surfaces following the standardized LCZ framework. Three classes—LCZ A (Dense Trees), LCZ B (Scattered Trees), and LCZ D (Low Plants)—were reclassified as DenseTree, ScatteredTree, and LowPlant and used to represent distinct vegetation types. All landscape metrics were computed separately for these categories within each 0.03° × 0.03° (~3 km × 3 km) grid cell.

To characterize the spatial structure of green spaces, three landscape metrics were calculated for each LCZ-based vegetation type: PLAND, ENN, and LSI. These metrics were selected for their ability to capture vegetation abundance, spatial configuration, and morphological complexity—three key dimensions that jointly influence microclimatic regulation. Specifically, PLAND reflects the overall vegetation coverage, which directly affects evapotranspiration and surface energy balance; ENN measures the degree of fragmentation or connectivity among green patches, with closer proximity facilitating more coherent cooling zones; LSI quantifies the shape irregularity of vegetation patches, which may influence edge exposure and heat dissipation efficiency. This combination of metrics has been widely adopted in urban climate and landscape studies to assess vegetation–temperature interactions under varying morphological contexts [41].

In addition to vegetation features, the LCZ dataset also supported the construction of several control variables—such as the proportion of compact or industrial built-up areas—which were incorporated to isolate the vegetation–compactness interaction effects. The LCZ dataset has been extensively used in urban climate research and offers a reliable basis for cross-city comparisons [42,43].

#### 4.2.3. POI Dataset: Functional Clustering and MIXD Construction

To quantify compact urban development and functional land-use intensity, the MIXD index was constructed based on POI data obtained from OpenStreetMap (OSM). This dataset contains geo-located entries for urban functions across a wide range of categories, including education, retail, healthcare, transportation, leisure, and administration [44]. An adaptive clustering approach based on the KANN-DBSCAN algorithm was employed, integrating k-nearest neighbor (KANN) analysis with density-based spatial clustering (DBSCAN). This method enables the spatially flexible identification of high-density functional clusters [45]. For each POI, the neighborhood radius (Eps) was estimated by averaging distances to its k-nearest neighbors, while the minimum points threshold (MinPts) was determined using an expectation-based approach that adapts to the overall POI distribution. Clusters containing three or more distinct POI categories were classified as mixed-use functional zones (MFZs). The density of MFZs within each 0.03° × 0.03° grid cell was subsequently calculated to derive the MIXD index, thereby capturing both spatial intensity and land-use diversity.

This approach reflects not only the spatial compactness of urban form but also the functional heterogeneity that characterizes mixed-use development—two essential dimensions of compact urban morphology. Compared to traditional metrics such as floor area ratio (FAR) or land occupancy index (LOI), which focus solely on built density, MIXD provides added value by capturing dynamic land-use interactions that influence microclimatic performance. Prior studies have demonstrated strong correlations between MIXD-like indices and established compactness metrics, lending empirical support to its use [35,46]. The resulting MIXD index thus serves as a theoretically grounded and empirically validated measure of compact mixed-functional development, suitable for spatial interaction analysis with green infrastructure.

#### 4.2.4. Supporting Datasets for Environmental and Socioeconomic Control

To account for additional factors that may influence urban thermal conditions, this study incorporated a set of ancillary datasets reflecting environmental features, urban structure, and demographic characteristics. Water-related variables were derived from high-resolution land cover data, including the percentage of water surface within each grid cell (pland_water) and the Euclidean distance to the nearest water body (enn_water), both of which help capture the spatial cooling influence of urban water bodies. Built-environment types were identified using LCZ classifications, focusing on five non-vegetation categories—sparsely built, compact low-rise, compact mid-rise, compact high-rise, and industrial zones—with their respective proportions calculated within each analytical grid. Urban structural intensity was measured through POI density (den_poi) and road density (road_den, km/km^2^), representing levels of functional activity and potential ventilation capacity. Socioeconomic characteristics were also considered, including population density (pop_den), SNS-based check-in intensity (inten_act) as a proxy for daytime mobility, and the proportion of elderly residents (ratio_eld), which may influence both thermal exposure and green space usage patterns. All supporting variables were spatially aligned to the 0.03° × 0.03° grid system to ensure consistency across datasets and enhance the robustness of multivariate analyses.

### 4.3. Variables and Indicators

This study constructed a structured analytical framework incorporating dependent, independent, moderating, and control variables to explore how compact urban development affects the cooling performance of green space. Urban heat was measured through three key metrics: UHI occurrence (binary), UHI area (km^2^), and UHI intensity (°C), representing the presence, extent, and severity of localized thermal anomalies, respectively.

Vegetation characteristics were quantified using three landscape metrics—PLAND, ENN, and LSI—calculated separately for three LCZ-based green space types: densely planted trees (DenseTree), scattered trees (ScatteredTree), and low-lying vegetation (LowPlant). These indicators allowed for a detailed assessment of spatial vegetation structure.

The moderating variable, MIXD, captured land-use compactness based on POI clustering, reflecting both functional diversity and development intensity. By introducing interaction terms between MIXD and vegetation metrics, the analysis identifies how compact form conditions may enhance or suppress vegetation-based cooling.

To control for external influences, four categories of ancillary variables were included: hydrological features (water coverage, proximity to water), LCZ-based built types, urban infrastructure (POI and road density), and socioeconomic attributes (population density, mobility, and aging ratio). These variables were incorporated to isolate the specific effects of vegetation and urban compactness on thermal outcomes. To ensure temporal consistency, most datasets were collected for the year 2020. For variables unavailable for that year—such as LCZ-based green space classifications and UHI metrics—the closest available time point, namely late 2019, was used. To verify the spatial validity of the dataset, global spatial autocorrelation tests using Moran’s I were conducted for all key variables. All test results were found to be statistically significant (*p* < 0.05), confirming strong spatial dependencies in both environmental and socioeconomic attributes. A complete list of variables is provided in Table 7.

### 4.4. Statistical Modeling Approach

To systematically assess how green space characteristics and compact urban development jointly shape the UHI effect, this study constructed a multi-layered analytical framework integrating classical regression and machine learning techniques. The modeling strategy was designed to address three core questions: whether green spaces reduce UHI occurrence, how they influence its spatial extent and intensity, and how such effects are moderated by land-use compactness.

First, logistic regression models were applied to estimate the probability of UHI presence, using binary outcome variables (0 = no UHI, 1 = UHI) for both daytime and nighttime conditions. This approach enabled evaluation of whether the spatial attributes of green spaces—PLAND, ENN and LSI—significantly reduce the likelihood of localized heat accumulation. The general form of the model is

(1) $$\log(\frac{P(Y=1)}{1-P(Y=1)})\;=\;\beta_{0}\;+\;\beta_{1}PLAND\;+\;\beta_{2}ENN\;+\;\beta_{3}LSI\;+\;\beta_{4}Control\;Variable$$

Second, ordinary least squares (OLS) regression models were employed to quantify the effects of the same explanatory variables on UHI area and UHI intensity (both continuous variables), offering insights into the spatial and thermal magnitude of heat stress. The linear model follows the specification

*Y* = *β*_0_ + *β*_1_*PLAND* + *β*_2_*ENN* + *β*_3_*LSI* + *β*_4_*Control Variable* + *ϵ* (2)

To investigate the moderating role of compact development, the baseline models were extended to include the MIXD variable (Mixed-use and Intensive Development) and its interaction terms with each green space metric. This allows testing for whether the effectiveness of vegetation in mitigating UHI is contingent upon surrounding land-use compactness, as follows:

*Y* = *β*_0_ + *β*_1_*PLAND* + *β*_2_*ENN* + *β*_3_*LSI* + *β*_4_*MIXD* + *β*_5_(*MIXD* × *PLAND*) + *β*_6_(*MIXD* × *ENN*) + *β*_7_(*MIXD* × *LSI*) + *β*_8_*Control Variable* + *ϵ* (3)

Finally, to capture nonlinear relationships and threshold effects, the study employed the eXtreme Gradient Boosting (XGBoost) algorithm, a tree-based ensemble learning method well-suited for modeling complex spatial interactions. Model interpretation was conducted using SHapley Additive exPlanations (SHAP), which quantify the marginal contributions of each predictor to the outcome variable. In addition, Partial Dependence Plots (PDPs) were used to visualize how MIXD values influence UHI metrics in a nonlinear manner and to identify key threshold points at which compactness begins to significantly alter vegetation cooling performance. Together, this integrative modeling framework provides both explanatory clarity and predictive depth, enabling robust interpretation of how green infrastructure interacts with urban form to regulate thermal environments under varying spatial conditions.

### 4.5. Research Workflow

This study follows an integrated analytical workflow that blends spatial statistics with machine learning to explore how compact urban development modulates the cooling performance of green spaces under different UHI conditions.

The process begins with the collection and preprocessing of multi-source urban datasets, including remote sensing imagery, POI-based land-use indicators, and urban climate classifications. All data were spatially aligned through rasterization and grid-based standardization to ensure consistency across analytical units. To understand the underlying structure of the variables, descriptive statistics and multicollinearity checks were conducted prior to modeling. Logistic regression models were then used to estimate the likelihood of UHI occurrence, while linear regression was applied to examine how green space characteristics influenced the spatial extent and thermal intensity of UHI. These statistical models established a foundational understanding of the associations between vegetation configuration and urban thermal environments. Building upon this baseline, interaction terms between green space metrics and MIXD were incorporated into the regression framework to assess how the effects of vegetation vary under different levels of land-use compactness. This allowed the study to evaluate not just whether green infrastructure cools the urban environment, but also under what conditions such cooling is amplified or constrained. To further capture nonlinear dynamics, the study employed the XGBoost algorithm, which is particularly suited to high-dimensional and interaction-rich data. SHapley Additive exPlanations (SHAP) were used to interpret variable contributions and identify key predictors, while Partial Dependence Plots (PDPs) helped visualize threshold effects—particularly where shifts in MIXD altered the effectiveness of green space interventions.

**Figure 4:**
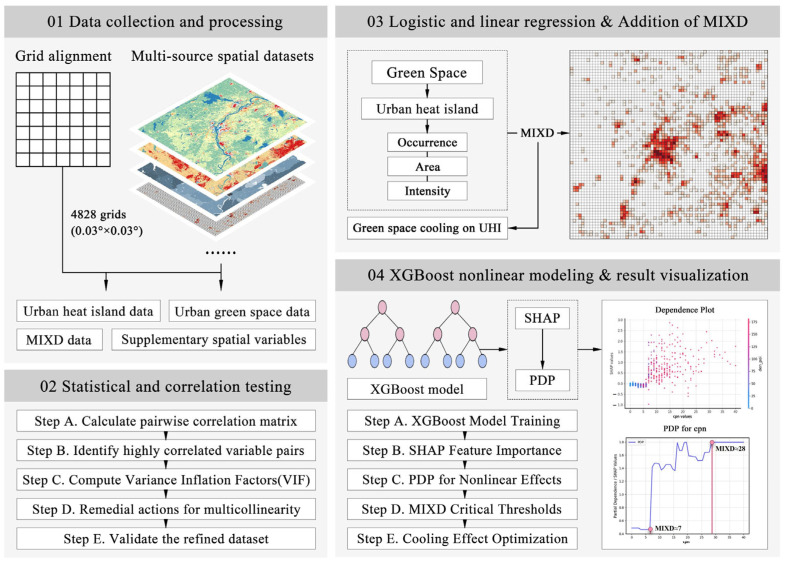
Research framework.

This combined workflow supports both hypothesis-driven inference and data-driven discovery, offering a replicable pathway for examining the spatial resilience of green infrastructure in compact, heat-vulnerable urban settings. A conceptual diagram of the research process is presented in Figure 4.

## 5. Conclusions

This study provides empirical evidence on how compact urban development modulates the thermal mitigation capacity of different vegetation types, based on a combination of interaction-based regression models and nonlinear machine learning analysis. By focusing on vegetation-specific responses across varying levels of land-use compactness, a landscape-informed understanding of urban greening strategies in dense environments is advanced.

Regression models reveal that compactness significantly alters the structure–function relationship of urban vegetation. For densely planted trees, increased compactness weakens their cooling effects through both spatial configuration and compositional factors; the interaction term between MIXD and patch aggregation shows a positive and significant effect on daytime UHI intensity (β = +42.15, *p* < 0.05), while increased landscape shape complexity also correlates with higher thermal intensity. These patterns suggest that densely vegetated areas, though effective under open conditions, may become heat-trapping under compact urban morphologies. In contrast, scattered trees demonstrate greater structural resilience. The interaction between MIXD and the proportion of scattered tree cover (PLAND) consistently yields negative and significant coefficients across all UHI metrics (e.g., β = –0.61 for daytime UHI area, *p* < 0.05), indicating that dispersed vegetative patches are less susceptible to the degrading effects of compactness. Low-lying vegetation presents a more mixed picture. While increased patch density (lower ENN) and shape simplicity (lower LSI) improve performance in compact environments, excessive coverage can lead to counterproductive effects. For example, MIXD × PLAND interactions become positive and significant for daytime UHI intensity (β = +0.00247, *p* < 0.05), implying that certain configurations of ground-level greenery may inadvertently amplify heat accumulation when compactness exceeds critical levels.

Building on these findings, the nonlinear analysis identifies threshold zones of MIXD beyond which vegetation cooling performance degrades sharply. For all three thermal metrics, MIXD values between approximately 7 and 15 represent a critical range where green infrastructure becomes increasingly less effective. Below MIXD ≈ 7, vegetation retains high cooling capacity, while beyond MIXD ≈ 28–37, thermal burdens plateau but remain elevated. These patterns suggest that there is no universally optimal level of compactness, but rather a context-dependent envelope within which vegetation-based cooling can be optimized. Taken together, the findings demonstrate that the interaction between urban form and green space is neither linear nor uniform. As such, urban densification strategies must account for both the structural configuration of vegetation and the morphological thresholds beyond which cooling benefits begin to deteriorate. Rather than focusing solely on the expansion of green coverage, planning efforts should consider how vegetation is spatially integrated into the urban fabric.

While the study leverages high-resolution spatial data and interpretable machine learning techniques, limitations remain. The analysis is based on a single city (Nanjing), and future research could extend the framework to multi-city comparisons, seasonal dynamics, and vertical greening structures. Additionally, more explicit climate simulation or scenario modeling could refine the actionable insights derived from observed patterns. Overall, this research contributes to a deeper understanding of how vegetation structure and urban compactness jointly shape urban climate resilience, offering a foundation for more adaptive and spatially nuanced greening strategies under densifying development pressures.

## Figures and Tables

**Table 1:** Logistic regression results for UHI occurrence during daytime and nighttime.

Variables	Model-1 (Exist_Day_uhi)	Model-2 (Exist_Night_uhi)
enn_densetree	−2375.2072 *	−1511.8080 *
enn_scatteredtree	8.5407	11.1037
enn_lowplant	−1007.8527 *	−599.6509 *
lsi_densetree	0.1608 *	0.1287 *
lsi_scatteredtree	−0.0972	−0.0395
lsi_lowplant	0.1021 *	0.0849 *
pland_densetree	−7.1435 *	−5.1989 *
pland_scatteredtree	−12.9973 *	−10.8915 *
pland_lowplant	−7.1370 *	−5.2738 *
Pseudo R-squared (R^2^)	47.26%	44.03%
LLR *p*-value	1.669 × 10^−283^	1.241 × 10^−253^
Converged	True	True

Note: The values in the table indicate the regression coefficients of the corresponding indicators, * represents significance *p* < 0.05. All models included a set of control variables but the results for these controls are omitted from the table for clarity and conciseness.

**Table 2:** Linear regression coefficients for UHI area and intensity across vegetation types.

Variables	Model-3 (Area_Day_uhi)	Model-4 (Area_Night_uhi)	Model-5 (Inten_Day_uhi)	Model-6 (Inten_Night_uhi)
enn_densetree	−124.735 *	−65.0188 *	−17.0815 *	1.4162
enn_scatteredtree	8.12278	12.6456 *	1.36792	0.9246 *
enn_lowplant	−36.6655	−20.1635	−10.9106 *	0.443587
lsi_densetree	0.0892258 *	0.0517599 *	0.0117965 *	0.000469431
lsi_scatteredtree	−0.131542 *	−0.069036 *	−0.016394 *	−2.28656 × 10^−5^
lsi_lowplant	0.01234	−0.00146468	0.00521127	−0.000524521
pland_densetree	−5.027 *	−2.65284 *	−0.715643 *	0.00501349
pland_scatteredtree	−4.46424 *	−2.27056 *	−0.648718 *	0.0451365
pland_lowplant	−4.85936 *	−2.57387 *	−0.654147 *	0.00773292
R-squared (R^2^)	68.85%	66.16%	47.06%	35.99%
Log likelihood	−9285.7	−8696.68	−979.318	−2500.06

Note: The values in the table indicate the regression coefficients of the corresponding indicators, * represents significance *p* < 0.05. All models included a set of control variables but the results for these controls are omitted from the table for clarity and conciseness.

**Table 3:** Logistic regression results showing the moderating effects of MIXD on UHI occurrence.

Variables	Model-7 (Exist_Day_uhi)	Model-8 (Exist_Night_uhi)
MIXD × enn_densetree	153.4405	363.2558 *
MIXD × enn_scatteredtree	−175.008	159.3026
MIXD × enn_lowplant	22.055	21.5943
MIXD × lsi_densetree	−0.0184 *	−0.0164 *
MIXD × lsi_scatteredtree	−0.0191	−0.0033
MIXD × lsi_lowplant	−0.0220 *	−0.0201 *
MIXD × pland_densetree	0.0036 *	0.0015
MIXD × pland_scatteredtree	−0.2438	−0.0125
MIXD × pland_lowplant	0.0226	0.0000239
Pseudo R-squared (R^2^)	47.19%	43.47%
LLR *p*-value	1.126 × 10^−300^	2.733 × 10^−250^
Converged	True	True

Note: The values in the table indicate the regression coefficients of the corresponding indicators, * represents significance *p* < 0.05. All models included a set of control variables but the results for these controls are omitted from the table for clarity and conciseness.

**Table 4:** Linear regression results showing interaction effects of MIXD on UHI area and intensity.

Variables	Model-9 (Area_Day_uhi)	Model-10 (Area_Night_uhi)	Model-11 (Inten_Day_uhi)	Model-12 (Inten_Night_uhi)
MIXD × enn_densetree	−28.8723	−22.8961	42.1481 *	-
MIXD × enn_scatteredtree	-	−26.7271	-	3.03447
MIXD × enn_lowplant	-	-	−22.3531 *	-
MIXD × lsi_densetree	0.00561704 *	0.00597969 *	0.000265582	-
MIXD × lsi_scatteredtree	−0.0333664 *	−0.0118443 *	−0.000648943	-
MIXD × lsi_lowplant	-	-	0.00127298 *	-
MIXD × pland_densetree	−0.00282488 *	−0.00165831 *	−0.000706301 *	-
MIXD × pland_scatteredtree	−0.613012 *	−0.194904 *	−0.0527385 *	-
MIXD × pland_lowplant	−0.00124231	−0.00159986	0.00247489 *	-
R-squared (R^2^)	70.66%	45.49%	67.87%	36.01%
Log likelihood	−9119.19	−1067.68	−8551.32	−2507.42

Note: The values in the table indicate the regression coefficients of the corresponding indicators, * represents significance *p* < 0.05. All models included a set of control variables but the results for these controls are omitted from the table for clarity and conciseness.

**Table 5:** Predictive performance of XGBoost models for different UHI metrics.

Model Target	MSE	RMSE	MAE	R^2^	Explained Variance
Daytime UHI Area	2.059	1.435	0.448	75.5%	0.755
Nighttime UHI Area	2.945	1.716	0.48	57.6%	0.577
Daytime UHI Intensity	0.099	0.315	0.108	36.9%	0.369

**Table 6:** Planning Strategies for Vegetation-Based Cooling under Varying Urban Compactness Levels.

MIXD Range	Thermal Response	Planning Strategies
Low (MIXD < 7)	Cooling performance stable; limited UHI amplification	Encourage moderate densification; protect large vegetated patches; use permeable materials
Moderate (9–28)	Rapid increase in UHI area and intensity; critical thermal threshold	Maintain vegetation connectivity; design ventilation corridors; promote rooftop and courtyard greening
High (MIXD > 37)	UHI effects plateau but cooling potential remains suppressed	Integrate vertical greening (walls, façades); implement 3D planting systems; require greening in building codes

**Table 7:** Variable description.

Variable	Content	Type	Moran’s I	Unit
exist_day_uhi	Daytime UHI occurrence (0 = No, 1 = Yes)	Dependent	/	Binary (0/1)
exist_night_uhi	Nighttime UHI occurrence (0 = No, 1 = Yes)	Dependent	/	Binary (0/1)
area_day_uhi	Area of daytime UHI	Dependent	0.6199 *	km^2^
area_night_uhi	Area of nighttime UHI	Dependent	0.6132 *	km^2^
inten_day_uhi	Intensity of daytime UHI	Dependent	0.4352 *	°C
inten_night_uhi	Intensity of nighttime UHI	Dependent	0.3920 *	°C
PLAND_DenseTree	Green space coverage ratio (Dense Trees)	Independent	0.6346 *	Percentage (%)
ENN_DenseTree	Euclidean nearest neighbor distance (Dense Trees)	Independent	0.1340 *	Meters (m)
LSI_DenseTree	Landscape Shape Index (Dense Trees)	Independent	0.6339 *	Dimensionless
PLAND_ScatteredTree	Green space coverage ratio (Scattered Trees)	Independent	0.6906 *	Percentage (%)
ENN_ScatteredTree	Euclidean nearest neighbor distance (Scattered Trees)	Independent	0.0715 *	Meters (m)
LSI_ScatteredTree	Landscape Shape Index (Scattered Trees)	Independent	0.5778 *	Dimensionless
PLAND_LowPlant	Green space coverage ratio (Low Plants)	Independent	0.7439 *	Percentage (%)
ENN_LowPlant	Euclidean nearest neighbor distance (Low Plants)	Independent	0.0993 *	Meters (m)
LSI_LowPlant	Landscape Shape Index (Low Plants)	Independent	0.4939 *	Dimensionless
MIXD	Mixed-use and intensive development index	Moderating	0.6045 *	Dimensionless
pland_water	Water body coverage ratio	Control	0.6329 *	Percentage (%)
enn_water	Nearest neighbor distance of water bodies	Control	0.1502 *	Meters (m)
lcz_sparselybuilt	Proportion of sparsely built-up area	Control	0.6328 *	Percentage (%)
lcz_cptlow	Proportion of compact low-rise buildings	Control	0.0716 *	Percentage (%)
lcz_cptmid	Proportion of compact mid-rise buildings	Control	0.1736 *	Percentage (%)
lcz_cpthigh	Proportion of compact high-rise buildings	Control	0.0662 *	Percentage (%)
lcz_indus	Proportion of industrial land	Control	0.1383 *	Percentage (%)
den_poi	POI density	Control	0.3632 *	Count/km^2^
road_den	Road density	Control	0.5204 *	km/km^2^
pop_den	Population density	Control	0.0476 *	Persons/km^2^
inten_act	Activity intensity (measured by check-ins)	Control	0.3906 *	Count

Note: * in the table denotes that the global Moran’s I value is statistically significant at the 5% level (*p* < 0.05).

## Data Availability

The data supporting the findings of this study are available from the corresponding author upon reasonable request.

## Abbreviations

The following abbreviations are used in this manuscript:

UHI	Urban Heat Island
MIXD	Mixed-use and Intensive Development
PLAND	Proportion of Landscape
ENN	Euclidean Nearest Neighbor distance
LSI	Landscape Shape Index
LCZ	Local Climate Zone
POI	Point of Interest
MFZ	Multi-functional Zone
